# Vaginal Resection of a Large Nascent Uterine Pyomyoma Following Late Miscarriage

**DOI:** 10.7759/cureus.98531

**Published:** 2025-12-05

**Authors:** Perrine De Walque, Philippe Van Trappen

**Affiliations:** 1 Gynecology, Vrije Universiteit Brussel, Bruges, BEL; 2 Gynecology/Oncology, AZ Sint-Jan Hospital, Bruges, BEL

**Keywords:** benign uterine tumor, fertility preservation, myoma nascent, uterine fibroid degeneration, vaginal myomectomy

## Abstract

Uterine myomas are common benign tumors in women of reproductive age and may undergo degenerative changes during or after pregnancy. In rare cases, rapid growth and necrosis can complicate the clinical course and pose significant risks for infection and fertility. We report the case of a 29-year-old nulliparous woman who presented two months after a late miscarriage at 15 weeks of gestation with severe abdominal pain and a necrotizing uterine fibroid. Imaging revealed a large type 2 myoma, initially classified as type 3, according to the International Federation of Gynecology and Obstetrics (FIGO) classification, with central necrosis, prolapsing through the cervix into the vagina. Despite broad-spectrum antibiotics, symptoms persisted, and inflammatory markers increased. A fertility-preserving vaginal myomectomy was performed, allowing complete removal of a 120 mm necrotic fibroid. Postoperative recovery was uneventful, and follow-up imaging at three months showed only a small stable intramural remnant. The uterine cavity appeared normal on hysteroscopy, and the patient remained asymptomatic. Necrotizing fibroids following miscarriage pose diagnostic and therapeutic challenges. Magnetic resonance imaging (MRI) provides essential information for surgical planning, and vaginal myomectomy can be a fertility-preserving alternative when the fibroid becomes nascent. This case illustrates that even FIGO type 3 fibroids can be effectively managed by vaginal myomectomy. The myoma may convert to a FIGO type 2 myoma under pregnancy-related hormonal stimulation and subsequent necrosis, and may become infected, forming a “pyomyoma nascent.” In selected patients, this approach enables rapid infection control, avoids hysterectomy, and preserves fertility.

## Introduction

Uterine myomas are the most common benign pelvic neoplasms in women of reproductive age [1,2]. They originate from the smooth muscle cells of the myometrium and can vary in size, number, and location. The International Federation of Gynecology and Obstetrics (FIGO) classification system categorizes fibroids from types 0 to 8, distinguishing submucosal, intramural, subserosal, and pedunculated variants based on their position relative to the uterine cavity [3]. Risk factors include early menarche, hereditary predisposition, Black ethnicity, arterial hypertension, and chronic stress [2,4-8]. Clinically, myomas may remain asymptomatic or present with abnormal uterine bleeding (which can lead to chronic anemia), pelvic pain, bulk-related symptoms (such as abdominal distension, bladder or bowel dysfunction, and early satiety), and reproductive issues (including infertility and recurrent pregnancy loss) [9].

During pregnancy, myomas may undergo morphological and vascular changes. Smaller fibroids (<1 cm) tend to grow more rapidly, while larger ones (>3 cm) often remain stable or even regress [10]. In some cases, rapid growth exceeds their vascular supply, leading to red degeneration-a hemorrhagic infarction characterized by ischemia, necrosis, and prostaglandin release [11]. This can cause severe abdominal pain, fever, and uterine irritability, occasionally mimicking obstetric emergencies [11]. Pyomyomas, although rare, may also develop [12-16]. These represent a suppurative transformation of a myoma secondary to bacterial infection, resulting in abscess formation, and manifest with a clinical presentation that closely parallels the symptomatology of red degeneration.

Here, we report a rare case of a large submucosal myoma, initially classified as a FIGO type 3 myoma, undergoing necrosis, infection, and partial expulsion following a pregnancy loss in the second trimester. The patient presented with systemic inflammatory signs and a necrotizing fibroid protruding through the cervix. Despite the potential need for hysterectomy, fertility was preserved through conservative vaginal myomectomy. This case highlights the diagnostic and surgical challenges in managing pyomyoma in the postpartum setting and illustrates the feasibility of uterus-sparing management in selected patients.

## Case presentation

Patient information

A 29-year-old woman with a medical history of hypothyroidism and endometriosis, with previous surgeries including an appendectomy and a diagnostic laparoscopy confirming superficial endometriosis. Her obstetric history includes two pregnancies with no live births (G2P0A2), consisting of one missed abortion and one spontaneous expulsion of a 15-week fetus following preterm prelabor rupture of membranes (PPROM) in December 2024. Last cervical cytology was normal. Current medications include levothyroxine (L-Thyroxine®) and an antagonist of gonadorelin (Ryeqo®).

Concerning the uterine myoma, ultrasound reports from various hospitals documented a FIGO type 3 myoma with a largest diameter of 24.5 mm in March 2023. By April 2024, it had increased to 50 × 50 × 32 mm. At the time of fetal expulsion in December 2024, the myoma measured approximately 80 × 70 mm. Three weeks later, it had further enlarged to 95 × 106 × 97 mm. The myoma was classified as a FIGO type 2 lesion.

Clinical findings

The patient was referred to our tertiary center because of the presence of a necrotizing uterine myoma and severe lower abdominal pain. At presentation, she reported ongoing suprapubic pain, especially in the evening, limited foul-smelling vaginal bleeding (red brown), urinary complaints, and normal bowel function. Clinical exam revealed a firm, enlarged uterus (~12-week size), suprapubic tenderness, and cervical motion pain. Parameters were stable. Transvaginal ultrasound demonstrated a 90 × 90 × 90 mm fundal intramural myoma that had progressed to a FIGO type 2 lesion, showing central necrosis extending toward the endometrial cavity. The endometrium measured 23 mm and appeared hypoechogenic, with intraluminal clots. Both ovaries were normal in appearance, and no free fluid was detected. The patient was admitted for intravenous piperacillin-tazobactam, oral doxycycline, and analgesia, with MRI and microbiology consultation planned.

Diagnostic assessment

Diagnosis of a necrotizing submucosal uterine myoma with red degeneration was confirmed by MRI, with an overlying infection suspected. The MRI demonstrated a large FIGO type 2 myoma with central necrosis. The myoma was bulging into the uterine cavity, protruding through the cervix, and extending into the upper vagina (pyomyoma nascent).

Microbiological analysis of intraoperative specimens revealed Escherichia coli as the causative pathogen. Laboratory testing was notable for elevated CRP, leukocytosis, and a progressive decline in hemoglobin levels, consistent with an inflammatory response and anemia of chronic disease. Despite clinical deterioration and limited response to broad-spectrum antibiotics, no signs of systemic sepsis were present.

Table 1 summarizes the patient’s laboratory results during hospitalization.

**Table 1:** Laboratory results over time / = test not performed

Date	Test		
	Hemoglobin (g/dL)	White blood cells (×10^9^/L)	C-reactive protein (mg/L)
22-02-25	9.3	13.9	238.6
24-02-25	8.8	16.6	238.4
26-02-25	8.0	11.8	189.9
28-02-25	/	15.8	110.5
01-03-25	/	13.5	54.9

Therapeutic intervention

Upon hospital admission, the patient was diagnosed with a necrotizing FIGO type 3 uterine myoma that had converted to a type 2, presenting with central liquefactive necrosis and overlying infection. Initial management included intravenous piperacillin-tazobactam, oral doxycycline, and analgesia, alongside close clinical and laboratory monitoring. Transvaginal ultrasound and gynecologic examination confirmed a tender, enlarged uterus with signs of endometrial clot retention.

An MRI revealed a 120 mm necrotic myoma, confirming the diagnosis of red degeneration with central liquefactive necrosis extending toward the endometrial cavity, suspected of an overlying infection (pyomyoma).

Due to persistent suprapubic pain and rising CRP levels despite broad-spectrum antibiotic therapy, the patient was transferred to our hospital. Given concern for progressing uterine infection and impending sepsis, intravenous meropenem was initiated.

A follow-up MRI revealed a myoma nascent bulging through the endometrial cavity and cervix into the proximal vagina.

Internal examination revealed that a necrotic myoma wall was clearly visible in the cervix with approximately 15 mm protruding into the vagina, accompanied by greenish-pink discharge without active red bleeding. Upon gentle traction with forceps, an additional 2 cm of myoma could be mobilized into the vagina, prompting a surgical approach.

**Figure 1:**
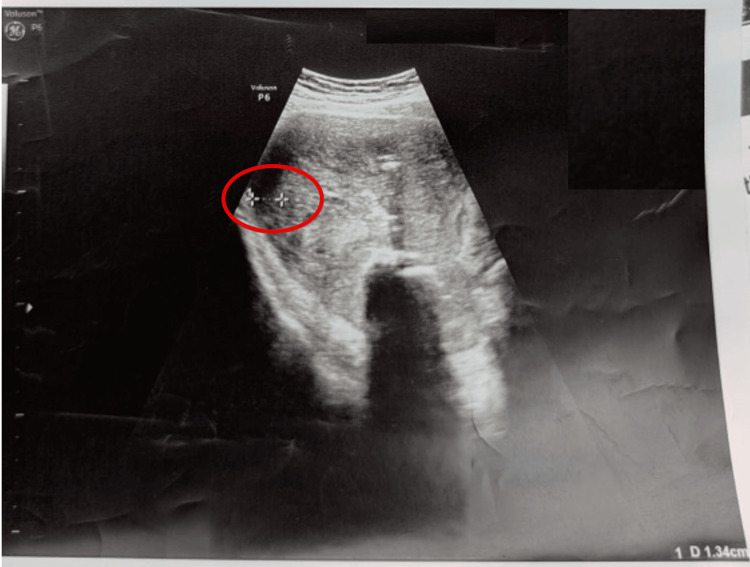
Intraoperative ultrasound shows the thickness of the uterine wall while removing the myoma nascent

The patient underwent a vaginal myomectomy using diathermic loop resection under abdominal ultrasound guidance, which helped prevent uterine inversion or perforation. The cervix was already dilated, and the myoma was in the process of expelling through the cervical canal. Therefore, further dilation was not required. Cervical dilation was approximately 15 mm. The procedure was performed with a ConMed® electrosurgical handpiece equipped with a medium loop electrode, set to 50-50 spray mode. At no point did the loop enter the intracervical canal. The myoma was grasped with a Kocher clamp to apply gentle traction, and a second Kocher was subsequently placed to maintain a secure grip during progressive resection. By applying controlled traction in this manner, we were able to resect fragments of the myoma while consistently maintaining a safe distance from the cervix. Figure 1 shows the intraoperative ultrasound demonstrating the thickness of the uterine wall during removal of the nascent myoma.

**Figure 2:**
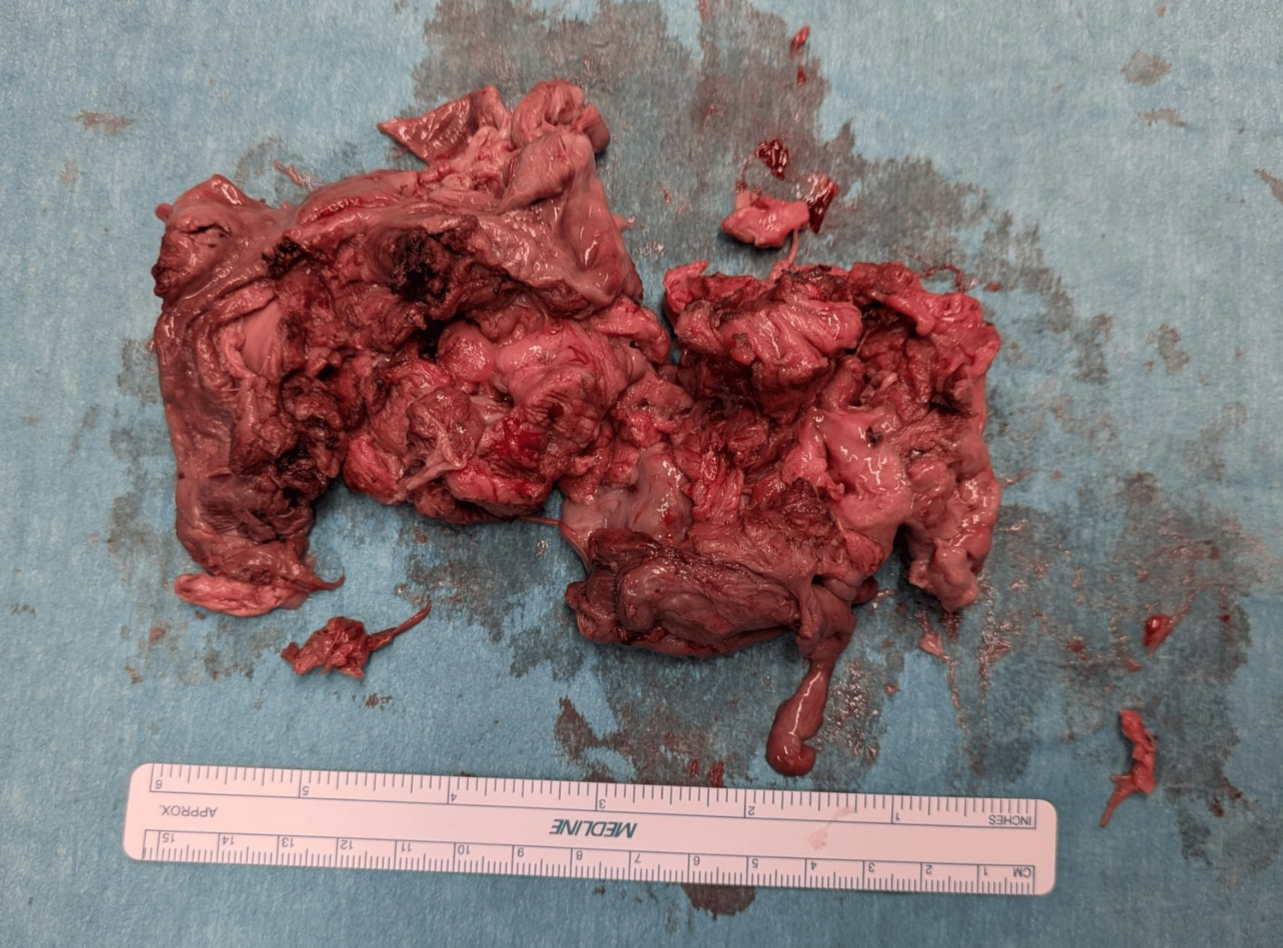
The extracted nascent myoma Histopathological analysis confirmed a necrotizing leiomyoma, composed of multiple fragments of spindle cell tissue without significant atypia and showing extensive tissue necrosis, consistent with a degenerating fibroid. Microbiological cultures of the excised tissue confirmed Escherichia Coli as the infectious agent.

Operative hysteroscopy could not be performed due to the large expelling myoma, which also obstructed the cervical canal. A 120-mm necrotic expelling myoma (myoma nascent), as shown in Figure 2, was successfully resected via the endocervical canal and vagina.

Intraoperative inspection revealed a grossly normal uterus and endometrial cavity. Blood loss was approximately 100 mL, but preventative tranexamic acid (Exacyl® 1 g IV) together with misoprostol (Cytotec® four tablets intrarectally) was administered, achieving effective hemostasis.

Microbiological cultures of the excised tissue confirmed Escherichia coli as the infectious agent.

Postoperatively, the patient continued treatment with oral amoxicillin-clavulanate (Augmentin®). Pain, leukocytosis, and CRP levels normalized during recovery. The patient was discharged four days after surgery.

Histopathological analysis, obtained two days after the patient’s discharge, confirmed a necrotizing leiomyoma, composed of multiple fragments of spindle cell tissue without significant atypia and showing extensive tissue necrosis, consistent with a degenerating fibroid.

Follow-up and outcomes

At the first outpatient follow-up, the patient reported a significant improvement in symptoms. Three months postoperatively, a pelvic MRI showed a small residual FIGO type 3 myoma located at the uterine fundus, measuring approximately 28.5 mm (AP) × 26.5 mm (transverse) × 24.9 mm (height). A diagnostic outpatient hysteroscopy revealed a normal uterine cavity, without any visible bulging of the remaining myoma. The endometrial lining appeared healthy, and both tubal ostia were visualized.

At the last gynecologic consultation, the patient was asymptomatic. The major necrotic mass had been completely removed, and only a small, stable intramural remnant remained. She continued hormonal therapy with an antagonist of gonadorelin (Ryeqo®) and reported no further pelvic pain, abnormal bleeding, or systemic symptoms.

**Figure 3:**
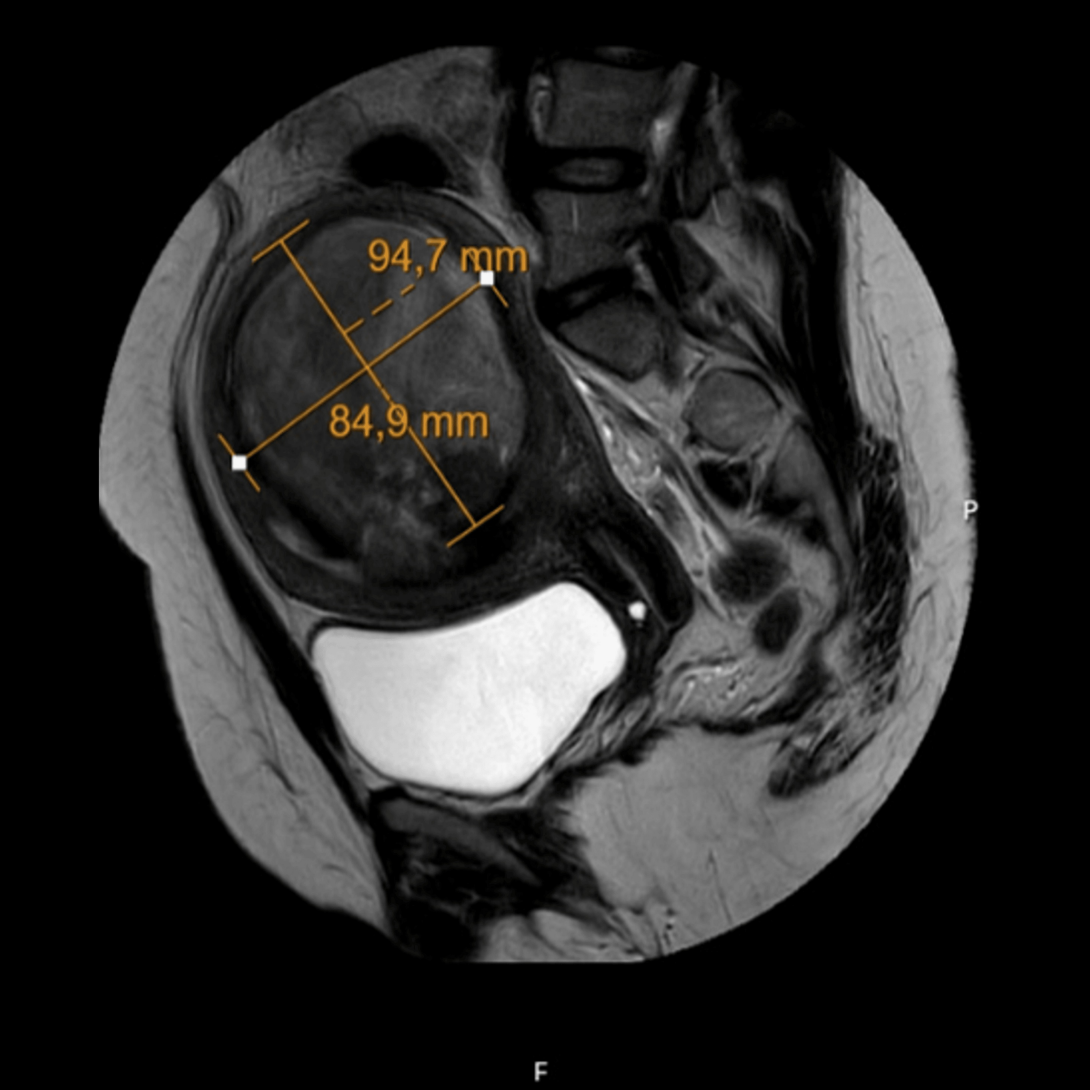
MRI six weeks after miscarriage

**Figure 4:**
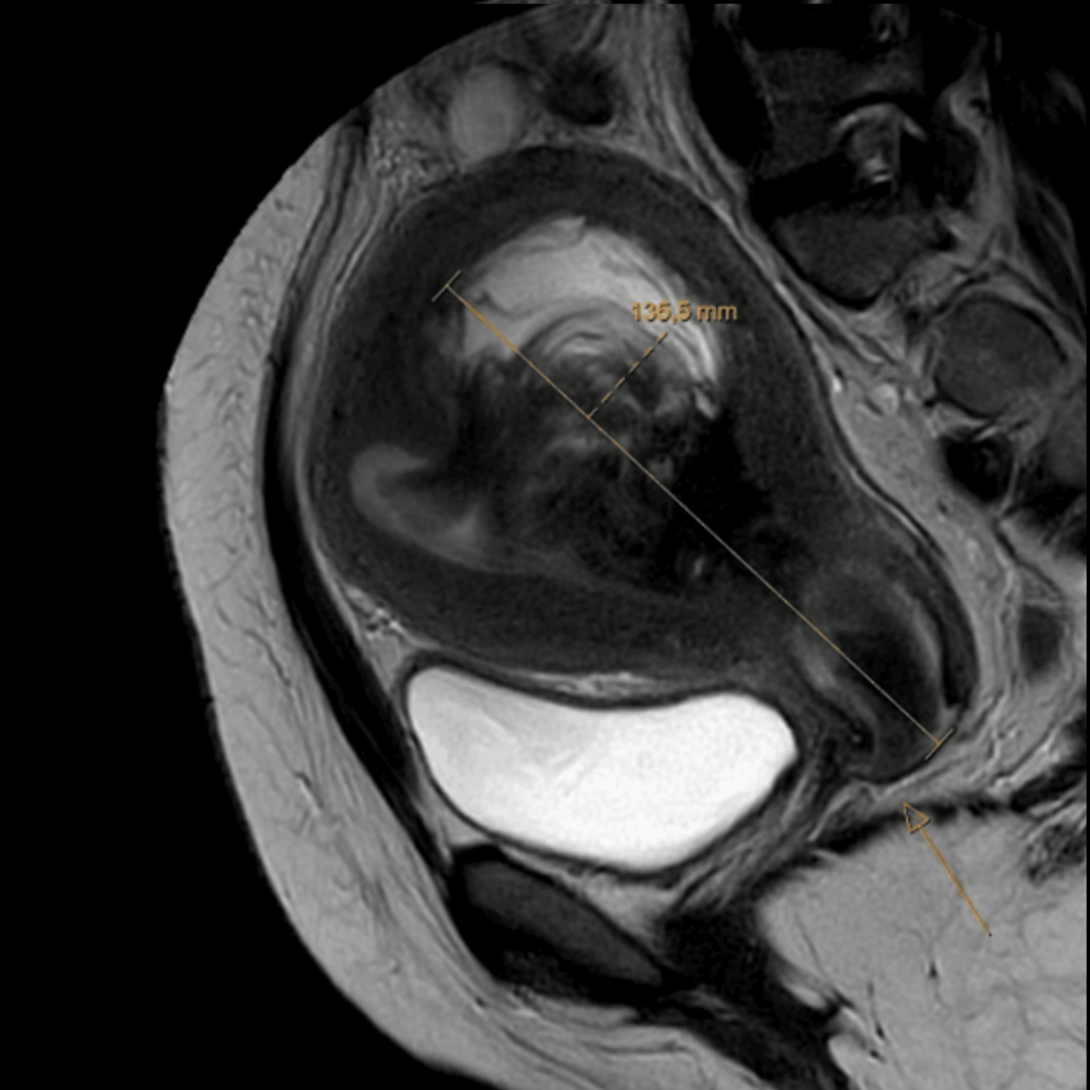
MRI of myoma nascent during hospitalization The arrow indicates the expelled portion of the nascent pyomyoma in the vagina. Known large heterogeneous T2-intermediate intense mass endocavitary within the uterine cavity, which is currently completely prolapsed into the endocervical canal and already protruding into the proximal vagina: differential diagnosis includes pedunculated endocavitary myoma versus endometrial polyp. The lesion is completely hypovascular. Spontaneous T1-hyperintense rim surrounding the lesion, compatible with endocavitary blood. Reactive enlarged lymph node in the left iliac region. Otherwise, no enlarged lymph nodes. No evidence of abscess formation in the abdomen.

**Figure 5:**
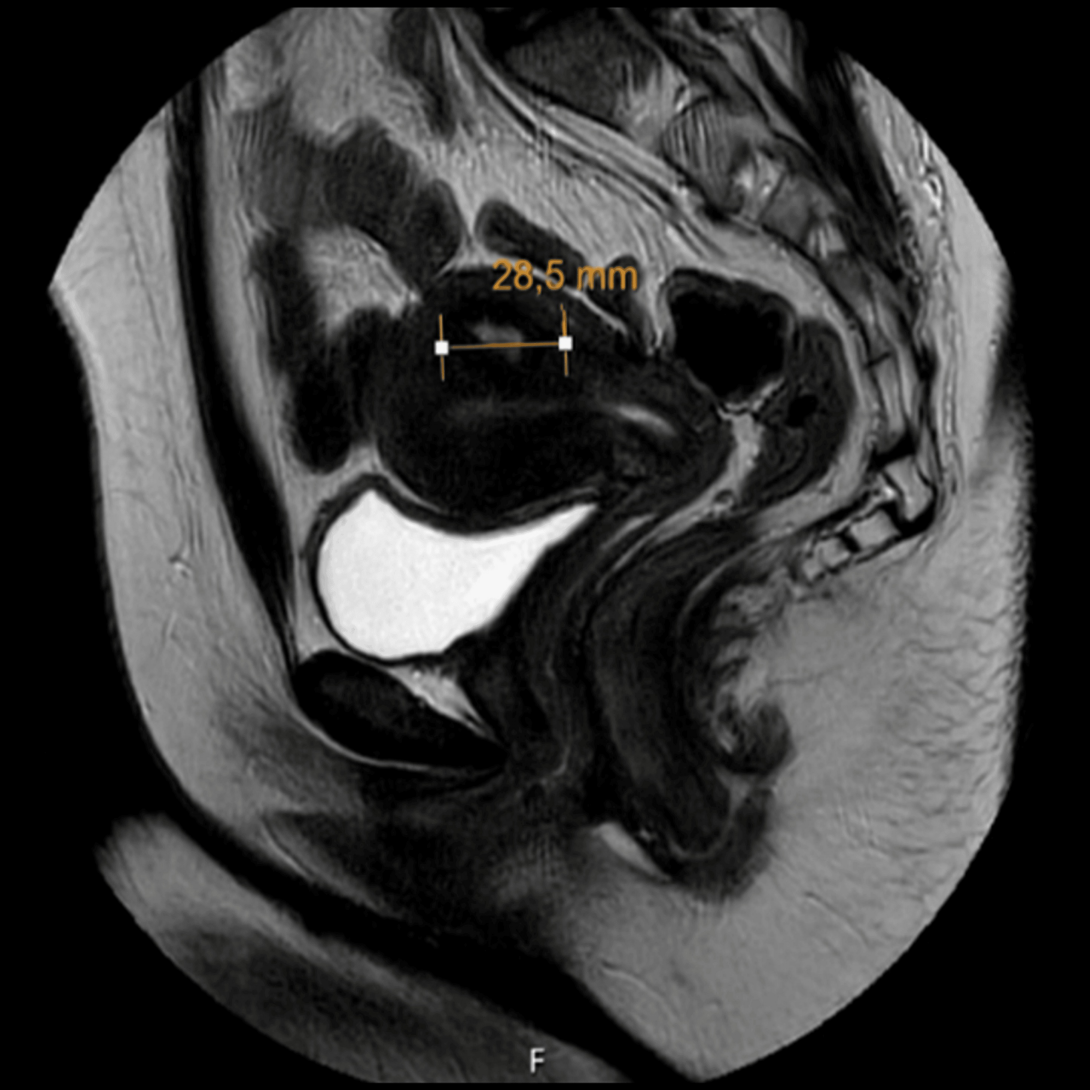
MRI at three months follow-up

Serial MRI images illustrate the progression of the case: six weeks after the late miscarriage (Figure 3), during hospitalization, demonstrating vaginal protrusion (Figure 4), and at the three-month follow-up (Figure 5).

## Discussion

This case report highlights the complex diagnostic and therapeutic challenges associated with a rapidly enlarging, necrotizing uterine fibroid complicated by infection following second-trimester fetal loss. The clinical course demonstrates how structural uterine pathology, particularly a FIGO type 3 myoma, can serve as both a mechanical and infectious risk factor in pregnancy and the postpartum period.

The strength of this case lies in the detailed longitudinal follow-up, including sequential imaging and direct visualization of the uterine cavity. The diagnostic accuracy provided by MRI was crucial in differentiating between degenerative change and potential abscess, and in demonstrating the progressive descent of the myoma into the cervix and vagina. Additionally, the interdisciplinary approach, including microbiology-guided antibiotic adjustment and minimally invasive surgical removal, highlights an effective fertility-sparing strategy in a complex, infection-prone setting. Although vaginal myomectomy is uncommon for a FIGO type 3 myoma, it proved feasible and definitive in this case. The myoma likely transitioned from FIGO type 3 to type 2 under hormonal stimulation during pregnancy, subsequently underwent necrosis, and progressed to a nascent expulsing lesion following a second-trimester miscarriage. This anatomical transformation enabled a vaginal approach, supported by intraoperative ultrasound and conservative hemostatic techniques.

However, several limitations are associated with this case. As a single-patient report, its findings cannot be generalized without caution. The infectious pathway, though likely ascending from post-abortive uterine exposure, cannot be confirmed with certainty. A causal relationship between the fibroid and pregnancy loss remains speculative, although the dramatic size increase and its proximity to the endometrial cavity support a pathophysiological link. Literature suggests that fibroids distorting the uterine cavity (particularly FIGO types 0-3) are associated with increased miscarriage rates and adverse pregnancy outcomes due to impaired implantation, placental separation, or infection [1,3,11]. Rapid growth during pregnancy may lead to ischemia and necrosis (so-called red degeneration), predisposing the fibroid to secondary infection by enteric or urogenital flora (becoming a pyomyoma) [2,12-18]. Furthermore, the lack of histopathological data at the time of discharge initially impeded full characterization of the myoma subtype and degree of degeneration.

Management of necrotizing or infected fibroids (pyomyoma) is not well standardized. Although conservative treatment with antibiotics is initially preferred, failure to respond, as in this case, necessitates surgical intervention. While laparoscopic or abdominal myomectomy remains the standard for most type 3 myoma, literature supports vaginal resection in selected patients in case the fibroid is prolapsing, as “myoma nascent” [18,19]. This approach offers reduced morbidity and faster recovery while preserving uterine integrity, especially relevant in patients with future fertility aspirations.

## Conclusions

This case demonstrates that a FIGO type 3 myoma, likely converted to type 2 under hormonal stimulation during pregnancy and becoming necrotic, infected, and expulsing (pyomyoma nascent) after second-trimester miscarriage, can be successfully managed through vaginal myomectomy with preservation of fertility. Timely imaging, surgical adaptability, and interdisciplinary management are essential when conservative treatment fails. Even infected, large necrotic fibroids with partial intracavitary extension can be addressed vaginally in selected cases, avoiding more invasive surgery.
